# Objects Mediate Goal Integration in Ventrolateral Prefrontal Cortex during Action Observation

**DOI:** 10.1371/journal.pone.0134316

**Published:** 2015-07-28

**Authors:** Mari Hrkać, Moritz F. Wurm, Anne B. Kühn, Ricarda I. Schubotz

**Affiliations:** 1 University of Münster, Institute of Psychology, 48149, Münster, Germany; 2 Max Planck Institute for Neurological Research, 50931, Cologne, Germany; 3 University of Trento, Center for Mind/Brain Sciences, Via delle Regole, 101, 38100, Mattarello (TN), Italy; University G. d'Annunzio, ITALY

## Abstract

Actions performed by others are mostly not observed in isolation, but embedded in sequences of actions tied together by an overarching goal. Therefore, preceding actions can modulate the observer's expectations in relation to the currently perceived action. Ventrolateral prefrontal cortex (vlPFC), and inferior frontal gyrus (IFG) in particular, is suggested to subserve the integration of episodic as well as semantic information and memory, including action scripts. The present fMRI study investigated if activation in IFG varies with the effort to integrate expected and observed action, even when not required by the task. During an fMRI session, participants were instructed to attend to short videos of single actions and to deliver a judgment about the actor’s current goal. We manipulated the strength of goal expectation induced by the preceding action, implementing the parameter "goal-relatedness" between the preceding and the currently observed action. Moreover, since objects point to the probability of certain actions, we also manipulated whether the current and the preceding action shared at least one object or not. We found an interaction between the two factors goal-relatedness and shared object: IFG activation increased the weaker the goal-relatedness between the preceding and the current action was, but only when they shared at least one object. Here, integration of successive action steps was triggered by the re-appearing (shared) object but hampered by a weak goal-relatedness between the actually observed manipulation. These findings foster the recently emerging view that IFG is enhanced by goal-related conflicts during action observation.

## Introduction

To observe an actor is not to understand his action. We can see his movements and recognize the objects he manipulates, but understanding means to know which goal, i.e., the final end state of the action, the actor is trying to achieve [1]. Interestingly, a correct understanding often establishes *before* the goal achievement, as the observer builds up expectations of goals that are deemed most probable against the backdrop of the current action's stage (a given time point during the unfolding of the action) and the observer's knowledge about typical actions (“scripts” (cf. [2,3])). However, we do not know much about how the observer’s brain integrates observed action stages and script knowledge to make sense of observed actions.

As there is a remarkable overlap between the networks underlying action execution and those underlying action observation [4], one may reason that the functional organization underlying the selection and control of our actions should also rule interpretation of observed actions. As actors, we integrate our current action's stage and our script knowledge to control our own actions. According to Koechlin and Summerfield [5], there are different hierarchical levels of control. Beyond simple stimulus-response associations, the current context of a stimulus is relevant for action selection, implementing so-called contextual control, i.e., the available objects (e.g. tomatoes, knife) are manipulated under the condition of a certain context (e.g. kitchen). On the next level, preceding events are considered, implementing so-called episodic control (e.g., the tomatoes have been washed already; therefore, the next action step can be executed, e.g. they can be sliced). The use of episodic information can be crucial for action selection, as a preceding event can demand a specific action that would not be performed otherwise [6], e.g. dumping a tomato after cutting it and noticing that it had gone off inside.

According to Koechlin and Summerfield [5], these different (and further) levels of control are suggested to map onto the ventrolateral prefrontal cortex (vlPFC), with lower control levels being reflected in more posterior regions than higher ones. Specifically, *contextual* control is reflected in posterior vlPFC, while *episodic* control is reflected in the anterior vlPFC. Nested control levels are suggested to impinge top-down on posteriorly adjacent premotor areas and their connections to the parietal cortex, thereby continuously shaping the sensorimotor action level, which controls the body’s concrete movements.

Applying these functions to action observation, we hypothesized that anterior vlPFC may figure in the integration of episodic information not only in the service of action control but also in the service of action perception. According to this assumption, anterior vlPFC should indicate difficulties with integration induced by episodic conflicts. Indeed, we found Brodmann Area 45 to be active when the observed actor performed actions that did not pertain to a common, overarching goal [7]. Here, episodic information induced a conflict as the previous encounter with the same actor influenced the interpretation of his current action. Likewise, activity in BA 45 decreases with the ease observed actions are integrated into action sequences cohered by a common goal [8]. These findings encourage the view that BA 45 engages in episodic integration as required during action observation.

The present fMRI study aimed to scrutinize the role of the BA 45 in integrating observed actions with the actions' immediate episodic history. To this end, we parametrically manipulated the goal-relatedness between consecutively presented actions (factor goal-relatedness). If BA 45 figures in episodic integration of observed actions, an action preceded by a weakly goal-related action should induce stronger activity in BA 45 than an action preceded by a strongly goal-related action. We therefore tested as first hypothesis that activity in BA 45 co-varies negatively with the goal-relatedness of consecutive actions.

**Fig 1:**
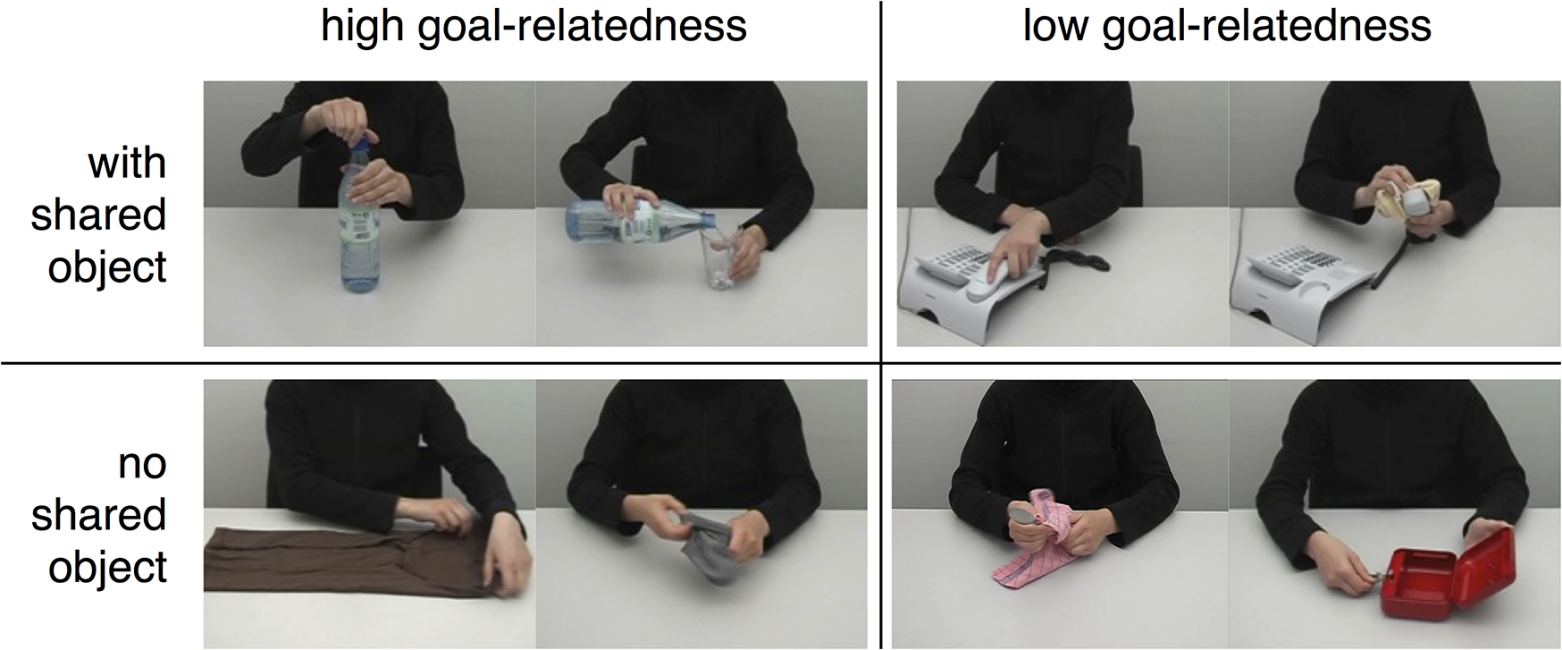
Snapshots of exemplary action videos. Action video pairs were assigned a parametric value of goal-relatedness as assessed in subjects in a post fMRI session survey. The parametric value of goal-relatedness of consecutively presented actions ranged from low (right column) to high (left column). Independent of this factor, videos shared a common object (upper row) or not (lower row). Examples show the following actions (in reading direction, from upper left): opening a bottle of water; pouring in a glass of water; grasping a telephone receiver; cleaning a telephone receiver; folding laundry (t-shirt); turning socks inside out; drying the dishes (spoon); unlocking a petty cash.

Two actions pertaining to an overarching goal often involve at least one common object [8]. Therefore, the tendency to integrate two consecutive actions under a common goal should be particularly encouraged for actions that share at least one common object. We implemented the two-level factor shared object (yes, no), to test the second hypothesis that the effect of goal-relatedness in BA 45, as postulated by the first hypothesis, should be more pronounced for actions that share a common object than for those that don't (Fig 1).

In a first step of analysis, we tested the effect of goal-relatedness separately for actions with at least one object shared with the preceding action and for actions without a shared object. According to Koechlin and Summerfield's [5] proposal and previous studies [7,8,9] we hypothesized BOLD response in BA 45 to be negatively correlated with goal-relatedness in either parametric contrast. Moreover, we expected this effect to be boosted in actions with shared objects. In a second step, we therefore calculated an interaction contrast between goal-relatedness and shared object.

## Methods

### Participants

Twenty-one right-handed, healthy and naïve volunteers participated in the study. Four participants had to be excluded from the analyses due to head motion artifacts that could not be removed by the motion correction procedure (N = 1) or self-reported phases of sleep (N = 3) during the experiment, resulting in 17 participants (9 females, 21 to 28 years, mean age 24.5 years). Participants were informed about potential risks of magnetic resonance imaging and screened by a physician. All participants gave informed written consent to participate in this study. The study was approved by the local ethics committee of the University of Cologne and was in accordance with the Declaration of Helsinki.

### Stimuli

**Fig 2:**
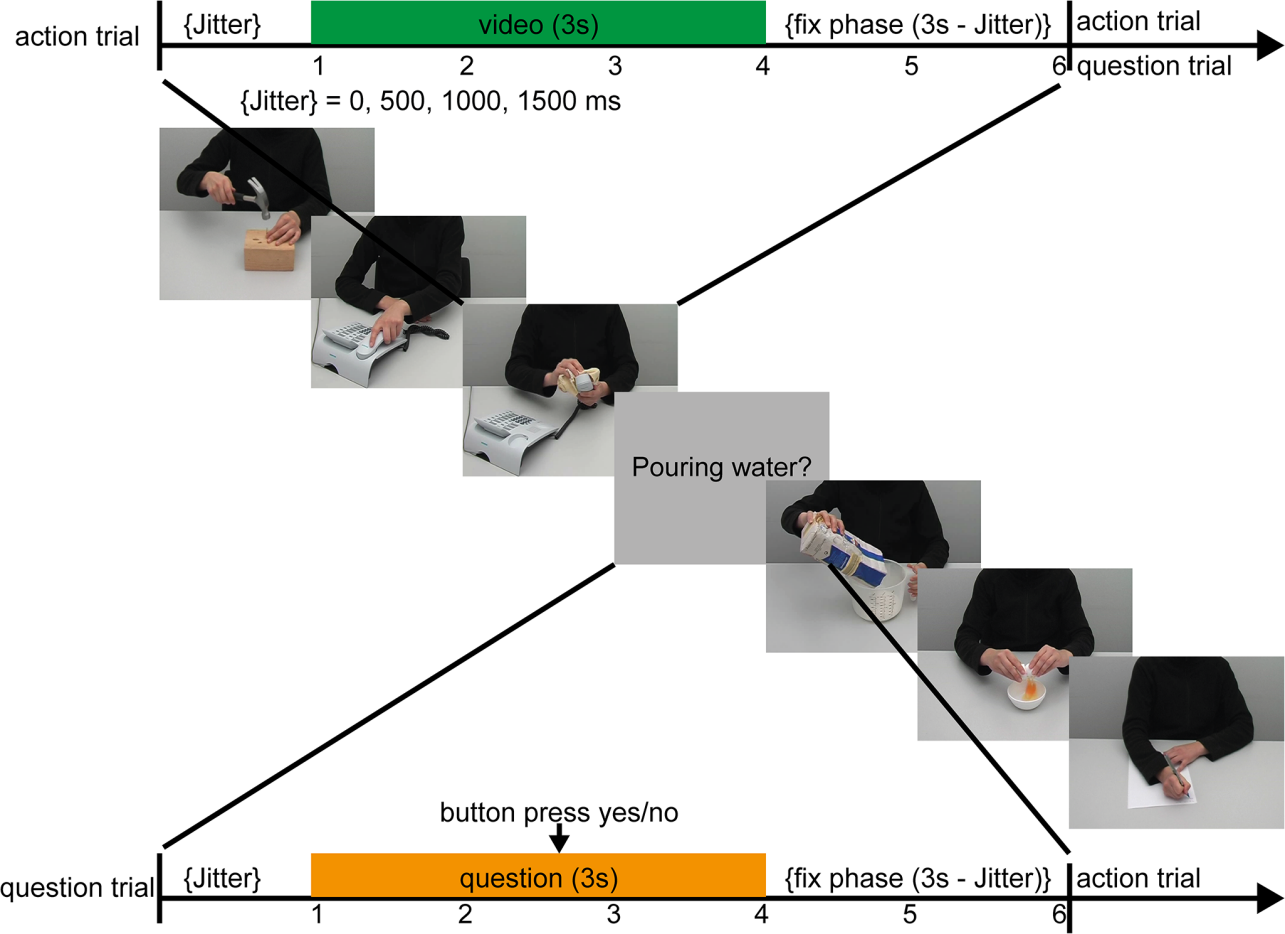
Event-related design (extract from the experimental time course). Action videos were presented intermixed with verbal action descriptions (question trials, 20% of action trials). Participants were instructed to attend to the videos and recognize the actions. In question trials participants had to indicate per button press whether an action description matched the preceding action (50%) or not (50%).

During the scanning session participants were presented with 254 video clips showing actions (action trials) and with 50 written action descriptions referring to these actions (question trials) (presentation software: Presentation Version 13.1, Neurobehavioral Systems, Albany, USA; visual stimulation: OptoStim, medres–medical research GmbH, Cologne, Germany). Each trial (6 s) started with a video clip or a question (3 s) which was followed by a fixation phase (3 s) (Fig 2). To enhance the temporal resolution of the BOLD response, a variable jitter (0, 500, 1000, or 1500 ms) was added after the fixation phase.

Each video clip showed a single object-directed action performed by an actress sitting at a table (Fig 1). Each action involved at least one target object (e.g. an orange in “peeling an orange”) and up to two additional objects (e.g. a knife and a plate in “halving an orange”). The actress’s face was not visible in order to minimize cognitive effects of person perception.

Video clips were arranged such that the probability of occurrence in real life between actions of consecutively presented video clips was as variable as possible. That is, goal-relatedness between subsequent clips were more or less probable in daily life (parametric factor goal-relatedness). As goal-relatedness was treated as a subjective construct, based on own experiences and a normative judgement on semantic relation and coherence, we used a simple linear Likert scale approach to model corresponding BOLD effects, as it is typically done in fMRI on subjective rating values. goal-relatedness was established according to a pilot study in a population that was not participating in the subsequent fMRI study. In the pilot study, forty participants (34 females, 19 to 30 years, mean age 21.7 years) rated how often two consecutively presented actions are performed one after another in everyday life on a six point Likert scale running from 1 “very rarely” to 6 “very often”. Goal-relatedness scores were assigned to the second trial of these action pairs. For pseudorandom arrangement, sets were compared and subsequently video clips were selected and arranged for the fMRI session such that goal-relatedness varied. Goal-relatedness scores were assigned to one of two conditions (factor shared object): (1) goal-relatedness with shared object, i.e., at least one object is used in the current and the previous video clip (57 trials), (2) goal-relatedness without shared objects (197 trials; Fig 1). Note that not the pilot but individual ratings were used to compute the regressor of goal-relatedness. To this end, the rating procedure was repeated in the fMRI participants after the fMRI session. These individual ratings were used to establish the parametric values for modeling the corresponding BOLD responses (see MRI Data analysis). An outlier analysis revealed that trials without shared objects that had a mean average score of 1.2 were more often represented than two standard deviations from the mean. To correct the distribution, we linearly interpolated the number of trials from the numbers closest to the outlier. This was done by randomly removing trials with score = 1.2 (n = 21) until the number of trials equaled the interpolated value. Note that also the first trial and trials following questions were excluded from the parametric analysis because these trials were not directly preceded by an action (51 trials in total). The final rating scores were distributed across the Likert scale levels for trials with and without shared object as follows: Trials with shared object: 1.0–1.9: 1 trial, 2.0–2.9: 0 trials, 3.0–3.9: 8 trials, 4.0–4.9: 20 trials, 5.0–5.9: 28 trials. Trials without shared object: 1.0–1.9: 72 trials, 2.0–2.9: 18 trials, 3.0–3.9: 22 trials, 4.0–4.9: 12 trials, 5.0–5.9: 1 trial. Notably, the distributions were different for trials with and without shared object (higher scores for trials with shared object, lower scores for trials without shared objects). To test for putative effects of distribution difference, we investigated the main effect of trials with vs. without shared objects (see Results).

### Task

Participants were instructed to watch the video clips attentively. They were told that after some of the video clips an action description would appear that either corresponded to the content of the preceding video clip or not and that they were to denote whether they accepted or rejected the description. Action descriptions (e.g., *pouring water*) did not amount to action-spanning (i.e., overarching) goals but referred only to each single action itself. Action descriptions were presented in a pseudo-randomized fashion mixed with the experimental trials. The response was given on a two-button response box, using the index finger to accept and the middle finger to reject the action description. Half of the action descriptions matched the object manipulation shown in the preceding trial, the other half did not. Error rates were analyzed to assess participants’ behavioral performance.

### MRI Data Acquisition

Imaging was performed on a 3-T Scanner (Siemens Magnetom TRIO, Erlangen, Germany) equipped with a standard birdcage head coil. Participants lay supine on the scanner bed with their right index and middle fingers positioned on the appropriate response buttons of a response box (Current Design, Inc, Philadelphia, USA). Form-fitting cushions were used to prevent head, arm, and hand movements. Participants were provided earplugs to attenuate scanner noise. Twenty-eight axial slices (4 mm thickness; 0.6 mm spacing; 200 mm field of view; 64 x 64 pixel matrix; in-plane resolution of 3 x 3 mm) covering the whole brain were acquired using a single shot gradient EPI sequence (2 s repetition time; 30 ms echo time; 90° flip angle; 116 kHz acquisition bandwidth) sensitive to BOLD contrast. After functional imaging, 28 anatomical T1-weighted Modified Driven-Equilibrium Fourier Transform (MDEFT) images [10,11] were acquired. In different session, high-resolution whole brain images were acquired from each subject to improve the localization of activation foci using a T1-weighted 3D-segmented MDEFT sequence (128 slices; field of view 256 mm; 256 x 256 pixel matrix; thickness 1mm; spacing 0.25 mm).

#### MRI Data Analysis

After motion correction using rigid-body registration to the central volume (Siemens motion correction protocol, Siemens, Erlangen, Germany), fMRI data were processed with the software package LIPSIA [12]. A cubic-spline interpolation was used to correct for the temporal offset between the slices acquired in one image. To remove low-frequency signal changes and baseline drifts, a temporal high-pass filter with a cutoff frequency of 1/75 Hz was used. Spatial smoothing with a Gaussian filter of 5.65 mm FWHM was applied. A rigid linear registration with six degrees of freedom (three rotational, three translational) was performed to align the functional data slices with a 3D stereotactic coordinate reference system. The rotational and translational parameters were obtained on the basis of the MDEFT and the EPI-T1 slices to achieve an optimal match between these slices and the individual 3D reference dataset. The MDEFT volume dataset with 128 slices and 1 mm slice thickness was standardized to the Talairach stereotactic space [13]. The rotational and translational parameters were subsequently normalized by linear scaling to a standard size. The resulting transformation parameters were then used to transform the functional slices using trilinear interpolation, so the resulting functional slices were aligned with the stereotactic coordinate system, thus generating isotropic voxels with a spatial resolution of 3 x 3 x 3 mm (27 mm^3^). The statistical evaluation was based on a least-squares estimation using the general linear model for serially autocorrelated observations [14,15].

The design matrix was generated with a gamma function, convolved with the hemodynamic response function. Brain activations were analyzed in one onset regressor time-locked to onset of the video clips, and the analyzed epoch comprised the full duration (3 s) of the presented video clips. The design matrix contained 6 predictors: Two parametric regressors of goal-relatedness for both levels of the factor shared object (with, without), two regressors of the main effect for both levels of the factor shared object (with, without), a parametric regressor for the reaction time in question trials (max. 3 s) and a parametric regressor for object continuity. The latter regressor was included as a regressor of nuisance to control for a possible confound between goal-relatedness and the number of reappearing objects in two consecutive video clips. To this end, the number of common objects appearing in two consecutive video clips was counted and assigned to the second trial as a regressor of nuisance. All parametric regressors were *z*-scored and modeled linearly. A Gaussian kernel of dispersion of 4 s FWHM was applied on the model equation, including the observation data, the design matrix, and the error term, to account for the temporal autocorrelation [15]. Contrast images, i.e., beta value estimates of the raw-score differences between specified conditions, were generated for each participant. As the individual functional datasets were aligned to the same stereotactic reference space, the single-subject contrast images were entered into a second-level random effects analysis for each of the contrasts.

For the group analyses one-sample *t* tests were used across the contrast images of all participants that indicated whether observed single participants effects (beta estimates of a parametric regressor) were significantly distinct from zero. The *t* values were then transformed into *Z* scores.

To correct for false-positive results among all measured grey matter voxels, an initial voxel-wise *z* threshold was set to *z* = 2.33 (*p* = .01). Resulting z-maps were corrected for multiple comparisons using a cluster-size threshold of 611 mm^3^ and a significance level of *p* < .05 as obtained by Monte Carlo simulations [16].

### Post-fMRI Rating Study

In a post fMRI session survey, participants were presented with all video clips in the same order as in the fMRI scanner for rating of the goal-relatedness. Here, the same rating procedure was performed as in the pilot study. The mean goal-relatedness rating score per video was calculated and subsequently used as parameter in the fMRI analysis.

## Results

### Behavioral Results

Performance during the fMRI session was assessed by response times of correctly answered trials and the rate of incorrectly answered trials. Response times for trials with shared object (mean ± standard error: 1000 ± 66 ms) did not differ significantly from trials without shared object (969 ± 63 ms), t(16) = 1.27, p = .221. Average response time was 984 ± 64 ms. The average error rate was low (6.2 ± 1.6%) and it did not differ between trials with (6.5 ± 2.4) and without shared object (6.2 ± 1.5) significantly, t(16) = .017, p = .865.

### FMRI Results

In a first step, we tested the hypothesis that activity in the IFG is modulated as a function of ease in integrating observed actions with episodic information. To this end, we investigated the parametric effects of goal-relatedness separately for videos with and without shared objects.

**Fig 3:**
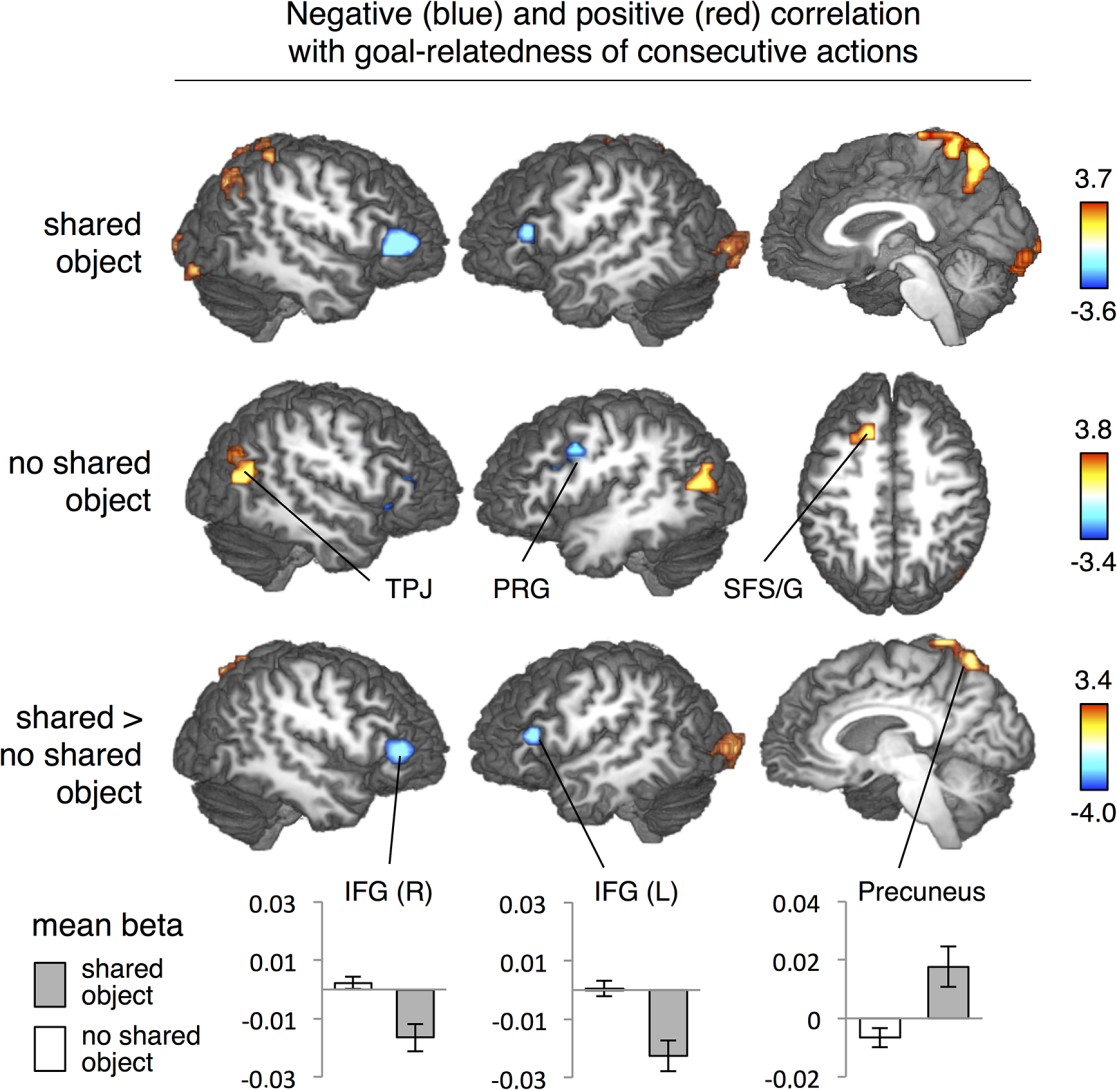
Areas that correlate negatively (blue) or positively (red) with the goal-relatedness of consecutively presented action videos. Upper row shows the parametric effect only for videos that shared at least one object with the preceding action. Middle row shows the same effect selectively for actions that did not share an object with the preceding action. The lower row depicts the interaction of these two parametric effects. Activation increased in bilateral IFG for weak goal-relatedness, but only when video clips contained a shared object. *Z*-thresholded at 2.33, corrected cluster threshold *p* = .05. IFG inferior frontal gyrus, AG Angular gyrus, aSFS/G anterior superior frontal sulcus/gyrus, PRG precentral gyrus, TPJ temporo-parietal junction.

The parametric analysis for goal-relatedness with shared object yielded activation in right IFG for weak goal-relatedness, i.e., the BOLD response negatively varied with goal relatedness (the BOLD response attenuated the stronger the action goals were related to each other). In addition, we found positive parametric effects of goal-relatedness in right precuneus, right angular gyrus (AG) and bilateral occipital gyrus (BA 18) for strong goal-relatedness (Table 1, Fig 3 upper row). In contrast, the parametric contrast for goal-relatedness without shared object did not reveal effects in the IFG, but in the right and left precentral gyrus (BA 6) as well as along the right anterior inferior frontal sulcus. Note that the latter activation had only 1 voxel overlap with the activation in the right IFG observed for trials with shared objects. We moreover found positive correlations between BOLD response and goal-relatedness in left anterior superior frontal sulcus and gyrus (aSFS; SFG), right AG, left temporo-parietal junction (TPJ), and left superior temporal sulcus (Table 1, Fig 3 middle row).

**Table 1:** Areas activated in the parametric analysis for goal-relatedness.

Anatomical Area	*x*	*y*	*z*	Z
***Parameter "goal relatedness" with shared object***				
**IFG (BA 45/47)**	-44	26	15	-3.19
	49	32	9	-3.59
**Precuneus**	10	-59	63	2.99
**Angular gyrus**	40	-61	48	2.83
**Occipital gyrus (BA 18)**	-23	-98	12	3.69
	13	-94	3	3.26
***Parameter "goal relatedness" without shared object***				
**Precentral gyrus (BA 6)**	-44	5	36	-2.94
	40	2	33	-3.41
**Inferior frontal sulcus**	40	32	12	-3.37
**Superior frontal sulcus/gyrus**	-17	26	42	3.40
**Temporo-parietal junction**	-41	-67	18	3.27
	37	-67	30	3.81
**Superior temporal sulcus**	-59	-4	-15	3.45
***Interaction Parameter "goal relatedness" with > without shared object***				
**IFG (BA 45/47)**	-44	26	15	-3.24
	49	35	9	-4.03
**Precuneus**	13	-67	54	2.85
**Occipital gyrus (BA 18)**	23	-95	12	3.39

Negative Z-scores indicate negative correlation. Macroanatomical specification, Talairach coordinates (*x*, *y*, *z*), and maximal Z scores (Z); corrected for multiple comparisons at *p* < .05.

Second, we tested the hypothesis that IFG shows negative parametric effects of goal-relatedness only when an object was shared. To this end, we calculated an interaction of the goal-relatedness and shared object. Here we found a negative interaction in bilateral IFG (pars triangularis, spreading into right pars orbitalis) indicating a specific negative parametric effect of goal relation in IFG for videos that share at least one object with the preceding video (Table 1, Fig 3 lower row). Positive correlating voxels were located in the right precuneus and right occipital gyrus.

Since the number of trials assigned to the parametric levels of the factor goal-relatedness were not perfectly balanced with regard to the levels of the factor of shared object, we finally investigated whether IFG was more activated by actions sharing at least one object with the preceding action (main effect of factor shared object: with vs. without) or parametrically correlated with the number of recurring objects (regressor of nuisance: object continuity). Neither contrast yielded activation in right or left IFG (see Table 2).

**Table 2:** Areas activated in the parametric analysis for object continuity (regressor of nuisance).

Anatomical Area	*x*	*y*	*z*	Z
***Parameter "Object continuity"***				
**Dorsal premotor cortex**	-26	-13	48	4.82
**Cerebellum**	16	-55	-36	3.68
**Inferior parietal lobule**	-59	-28	42	3.71
	52	-28	48	3.66
**Middle insula**	-38	-4	12	3.71
	37	5	-6	3.36
**Temporo-parietal junction**	-47	-67	21	-3.21
**Occipital gyrus (BA 18)**	-20	-91	15	-4.03
	13	-97	3	-3.89
**Hippocampus**	-29	-37	3	-3.33
**Fusiform gyrus**	22	-37	3	-3.15
***Shared vs*. *no shared objects***				
**Temporo-parietal junction**	-47	-70	21	4.48
**Lateral orbitofrontal cortex**	34	35	-12	3.33
**Occipital gyrus**	-23	-94	6	4.13
	10	-91	-3	4.03
**Superior frontal sulcus**	-29	17	45	3.78
***No shared vs*. *shared objects***				
**Dorsal premotor cortex**	-26	-16	48	4.38
**Supplementary motor area**	10	1	57	4.29
**Intraparietal sulcus**	52	-20	48	3.21
	-59	-23	42	2.91
**Supramarginal Gyrus**	-59	-20	24	3.50
	58	-17	30	3.26
**Frontal operculum**	-35	16	9	4.00
	37	22	3	3.75
**Cuneus**	-2	-77	30	2.81
**Cerebellar cortex**	16	-53	-39	3.72

Macroanatomical specification, Talairach coordinates (*x*, *y*, *z*), and maximal Z scores (Z); corrected for multiple comparisons at *p* < .05.

## Discussion

Ventrolateral prefrontal cortex is suggested to mediate the episodic embedding of ongoing action not only when executed [5] but also during observation [7, 8]. Here we used fMRI to investigate if activation in this area, particularly inferior frontal gyrus (IFG), varies as a function of the amount of episodic conflict induced by the action’s immediate history. To this end, we manipulated the strength of goal-relatedness between the preceding and the currently observed action as well as the presence of a shared object from the preceding action. Goal-relatedness was hypothesized to be negatively correlated with BOLD response in IFG, as weaker relation between goals of two actions was expected to induce a conflict for episodic integration, and even more so in actions that share one object with the preceding action.

Data showed that activity in bilateral IFG decreased the more an action’s goal was related to the preceding action, but only when a shared object was present. As we do not find a main effect of shared vs. no-shared object in the same anatomical area, it is unlikely that this interaction is driven by unequal distributions of goal-relatedness scores for trials with and without shared object. Hence, findings suggest that both the immediately preceding actions’ goals as well as the re-appearance of the previously manipulated object have a significant impact on the processing of observed action.

According to Koechlin and Summerfield [5], anterior vlPFC reflects episodic control, i.e., the consideration of preceding events when we control our own actions. This area has also been found activated when the integration of episodic information induced a conflict during action observation [7]. To resolve such a conflict, the retrieval of alternative goals and the selection of the goal that fits best are necessary. Likewise, activity in vlPFC decreases when semantic retrieval and selection are facilitated during the observation of consecutive actions that share a common overarching goal [8]. Such retrieval and selection mechanisms are thought to be mediated by the anterior vlPFC, specifically left IFG [17,18]. The present study specifies the IFG contribution to the effect that activity is only modulated when there is a shared object that binds two actions that do not match on the goal level. Goal-relatedness without shared object did not modulate activation in IFG. Hence, data imply that IFG was not constantly synthesizing episodic relations between successively observed actions, but only when there was an external trigger (here: a re-appearing object) to do so. There is also behavioral evidence for this, as infants do not link all actions in sequence but use further information [19].

A potential mechanism is that the re-appearance of the object that has been manipulated beforehand perpetuates this manipulation memory; this in turn may conflict with the actually observed action, as in our study. Since primed objects can be already recognized after 300 ms [20], this conflict may be induced even during the slower analysis of the unfolding manipulation.

Our findings particularly concur with a study on passive conceptual expansion necessary for creativity [21]. Authors reported bilateral IFG activation when participants thought about unusual but appropriate functions of objects. In such a case, an object is used to achieve a goal that is normally not related to this object, e.g., planting a flower in a shoe. Other studies indicate that IFG is sensitive to semantic associations and distance, with higher activity for weak associations [22] and larger distances [23]. Bunge and colleagues [22] asked their participants to make judgments about analogical and semantic relations between word pairs. Weak associative strength between word pairs (e.g. note-scale vs. bouquet-flower) led to both increased task difficulty and increased activity in IFG. Similarly, Green and colleagues [23] used an analogy rating. Semantic distance was assessed as the difference in meaning of two word pairs. Independent of task difficulty, greater semantic distance elicited stronger activity in IFG. In the present study, actions with weaker goal-relatedness were semantically less associated and more distant and a conceptual expansion might have been necessary to relate two actions associated by a shared object to an overarching goal that has a low probability.

Finding IFG to be particularly enhanced by a conflict on the goal level endorse recent studies in which we found that action performed in unusual rooms activated left IFG [9]. Here we argued that rooms modify the observer's expectation in view of the observed action's goal. As IFG increased for goal-incompatible compared to goal-neutral spatial contexts, IFG seems to reflect a conflict that is induced on the level of the expected goal. Moreover, in another fMRI study we found activity in left IFG was particularly enhanced when actions were performed by the same actor but not for a common, overarching goal [7]. Notably, this effect was neither driven by goal-incompatibility per se (as effects in IFG were absent for changing goals when performed by changing actors) nor was it driven by the re-appearance of the actor (as effects in IFG were absent for a continuous actor as long as he performed according to an overarching goal). Rather it seems that IFG was engaged because the shared actor implied a shared goal while actually changing goals were presented. In a similar vein, when actions shared a common overarching goal, IFG activity decreased as semantic integration of the observed actions became increasingly easier [8]. The present study corroborates and further extends these findings by showing that when consecutive actions share an object, this triggers the attempt to integrate them under a common goal. Together, these three studies and our present findings converge to the suggestion that IFG is engaged by a conflict between the expected goal (may it be implied by the room, the actor, or an object) and the goal the actually observed action aims at.

### Concluding Remarks

Action steps do not occur in temporal isolation; rather they make up semantically meaningful episodes integrated by overarching goals. Since integration of episodic as well as semantic information has been suggested to be mediated by lateral prefrontal sites, the present fMRI study explored the impact of the strength by which successive actions are bound by overarching goals. It shows that the inferior frontal gyrus (IFG) is tuned to the level of goal-relatedness between consecutive action steps, but only when they share at least one common object. Thus it seems that IFG plays a role in the detection and/or resolving of mismatch on the goal level: while the shared object implies continuity of the goal, the actual observed manipulation signals for a new goal. Together with recent studies, present findings endorse the view that IFG is engaged in action goal integration, triggered by the room, the actor, or–as in the current study–by objects.
