# Correction: Objects Mediate Goal Integration in Ventrolateral Prefrontal Cortex during Action Observation

**DOI:** 10.1371/journal.pone.0137813

**Published:** 2015-09-02

**Authors:** Mari Hrkać, Moritz F. Wurm, Anne B. Kühn, Ricarda I. Schubotz

The following information is missing from the Funding section: We acknowledge support by the Open Access Publication Fund of the University of Muenster.

The complete, correct Funding statement is: The Max Planck Institute financed the fMRI study (http://www.nf.mpg.de/). We also acknowledge support by the Open Access Publication Fund of the University of Muenster. The funders had no role in study design, data collection and analysis, decision to publish, or preparation of the manuscript.
